# What factors influence ward nurses’ recognition of and response to patient deterioration? An integrative review of the literature

**DOI:** 10.1002/nop2.53

**Published:** 2016-04-26

**Authors:** Debbie Massey, Wendy Chaboyer, Vinah Anderson

**Affiliations:** ^1^ Anaesthetics Department, Nambour General Hospital, Sunshine Coast Hospital and Health ServiceHospital RdNambour QLD 4560Australia; ^2^ Griffith University; ^3^ NHMRC Centre of Research Excellence in Nursing (NCREN)Menzies Health Institute Queensland School of Nursing and MidwiferyGriffith UniversityQLD 4222Australia; ^4^ Institute of Health and Care SciencesGothenburg UniversityAustralia; ^5^ NHMRC Centre for Research Excellence in Nursing Interventions for Hospitalised Patients (NCREN)Centre for Health Practice InnovationMenzies Health Institute Qld Gold Coast CampusQld 4222Australia

**Keywords:** Integrative review, nurses, patient deterioration, recognizing, responding

## Abstract

**Aim:**

In this integrative review, we aimed to: first, identify and summarize published studies relating to ward nurses' recognition of and response to patient deterioration; second, to critically evaluate studies that described or appraised the practice of ward nurses in recognizing and responding to patient deterioration; and third, identify gaps in the literature for further research.

**Design:**

An integrative review.

**Methods:**

The Cumulative Index to Nursing and Allied Health Literature (CINAHL) Ovid Medline, Informit and Google Scholar databases were accessed for the years 1990–2014. Data were extracted and summarized in tables and then appraised using the Mixed Method Appraisal Tool. Data were grouped into two domains; recognizing and responding to deterioration and then thematic analysis was used to identify the emerging themes.

**Results:**

Seventeen studies were reviewed and appraised. Recognizing patient deterioration was encapsulated in four themes: (1) assessing the patient; (2) knowing the patient; (3) education and (4) environmental factors. Responding to patient deterioration was encapsulated in three themes; (1) non‐technical skills; (2) access to support and (3) negative emotional responses.

**Conclusion:**

Issues involved in timely recognition of and response to clinical deterioration remain complex, yet patient safety relies on nurses’ timely assessments and actions.

## Background

The past decade has seen increased focus on recognizing and responding to deteriorating hospitalized patients (Australian Commission on Safety and Quality in Health Care (ACSQHC) 2010, Institute for Healthcare Improvement, 2008, National Institute for Health and Clinical Excellence (NICE), 2007). Much of this interest has been prompted by findings that demonstrated patient deterioration is often not recognized or responded to in a timely manner (Hodgetts *et al*. 2002, Jacques *et al*. 2006). Failure to recognize and respond to patient deterioration and escalate care has led to an increased risk of adverse events (AEs) in hospitalized patients that may have been avoided if patient deterioration had been recognized and responded to earlier (Massey *et al*. 2014). This integrative review first identifies the problems with recognizing and responding to clinical deterioration then describes the methods used in the review and our findings. Our analysis provides a contemporary understanding of the problems and issues in this area and potential research directions.

### Problem identification

There is a clear recognition of the frequency and adverse events in hospitals, with many studies and systematic reviews providing insights into the risks hospitalized patients face. For example, a systematic review of eight studies from the US, Canada, the UK, Australia and New Zealand, highlights that the median overall incidence of adverse events was 9·2% and almost half of these events were regarded as preventable (De Vries *et al*. 2008). More recently, Jha *et al*. (2013) conducted an extensive review of observational studies to estimate the burden of adverse events worldwide. They found that approximately 43 million adverse events occur each year around the globe and are responsible for 23 million associated disability‐adjusted life years, increasing hospital length of stay, decreasing quality of life and increasing morbidity and mortality (Vincent *et al*. 2001, Forster *et al*. 2003).

Nurses’ ability to recognize and respond to signs of patient deterioration in a timely manner plays a pivotal role in patient outcomes (Purling & King 2012) and preventing or minimizing major AEs. There is increasing awareness of the factors inhibiting nurses from escalating care for patients who deteriorate (Cox *et al*. 2006, Shearer *et al*. 2012, Massey *et al*. 2014). However, why ward nurses fail to recognize and respond to patient deterioration has not been extensively studied. There is clearly a need for a detailed and holistic analysis and synthesis of the relevant literature to elucidate the factors that contribute to ward nurses’ timely recognition of and response to patient deterioration. Critical analyses and syntheses of published international research is the focus of this integrative review. By exploring this complex clinical problem, gaps in knowledge and understanding of this important clinical topic will be illuminated and suggestions for future research will be proposed, potential solutions to improve clinical practice and improve patient outcomes will also be recommended. For this review, a deteriorating patient is defined as:

A patient who moves from one clinical state to a worse clinical state which increases their individual risk of morbidity, including organ dysfunction, protracted hospital stay, disability or death (Jones *et al*. 201, page, 1033).

The initial stage of a literature review requires a clear identification of the problem that the review is addressing (as described above) and the review purpose and aims (Whittemore & Knafl 2005). The aims of this integrative review were:

- Identify and summarize published studies relating to ward nurses’ recognition of or response to patient deterioration;
- Critically evaluate studies that describe or appraise the practice of ward nurses in recognizing and responding to patient deterioration; and
- Identify gaps in the literature for further research.

## Method

The integrative review method summaries and critiques literature on a clinical problem or phenomenon of concern and incorporates multiple perspectives and types of literature. Thus, the potential to contribute to a holistic understanding of a clinical problem is the hallmark of the integrative review. To enhance the rigor of the review, we used Whittemore and Knafl's systematic framework (Whittemore & Knafl 2005). Consistent with the framework, the stages of the review were: (1) Problem identification, as outlined in the introduction; (2) literature search; (3) data evaluation; (4) data analysis and (5) data interpretation and presentation of results.

### Literature search

Well‐defined literature search strategies are critical for enhancing the rigor of any type of review because incomplete and biased searches result in the potential for inaccurate results (Whittemore & Knafl 2005). In May 2014, three search strategies were employed to enhance the quality of this review (Whittemore & Knafl 2005), with search strategy one informing search two and three. With the assistance of a health librarian, a computerized database search of the Cumulative Index of Nursing and Allied Health Literature (CINAHL), PubMed and Medline was performed using a combination of various keywords and MeSH terms including patient deterioration, deterioration, pre‐arrest period, emergency assistance, vital signs, nurses, recognizing and responding. Table 1 contains this initial search strategy. The second search strategy involved hand‐searching reference lists of retrieved articles to find relevant literature not previously identified. Finally, the citations of retrieved articles were searched using Scopus to identify subsequent articles.

**Table 1:** First search strategy used via computerized databases

Steps	CINAHL Headings	Medline MeSH terms	PubMed Headings
S1	Recognition and response to deterioration and or vital signs	Patient deterioration OR deteriorating patient and OR vital signs	Patient deterioration and recognising and responding and vital signs.
S2	Nurses, OR Ward Nurses OR RNs OR inpatients	Nurses, OR Ward Nurses OR RNs OR Nursing	Nurses, OR Ward Nurses OR RNs OR Nursing
S3	Pre‐arrest period, emergency assistance, vital signs,	Peri arrest period, emergency assistance,	Peri arrest period and emergency assistance
S4	S1 and S2	S1 and S2	S1 and S2
S5	54	S4	S4
S4	Limiters: Date of publication: English Language. Narrowed by speciality: General wards.	Limiters: Date of publication: English Language. Narrowed by speciality: General wards.	Limiters: Date of publication: English Language. Narrowed by speciality: General wards.

### Inclusion and exclusion criteria

Consistent with integrative review methodology, there were no restrictions placed on research designs or study types. Only those studies that meet the following criteria were included: the studies had to focus on ward nurses' recognition and response to deterioration of the adult ward hospitalized patient, so studies that evaluated rapid response systems or track and trigger systems were excluded. Specialized areas like critical care, emergency and paediatrics were excluded because these clinical areas frequently use specialized equipment to monitor and survey patients at risk of deterioration. Ward nurses do not typically have access to this equipment or necessarily the skills required to use them. These specialized areas also have increased nurse: patient ratios that do not reflect the ward environment. Studies from 1999 and 2014 were included. The concept of the deteriorating ward patient has only recently emerged in the literature following McQuillan *et al*. (1998) seminal paper on suboptimal ward care and thus, it was important to capture work published after this work. Reviewing earlier would not capture contemporary healthcare practices.

### Search outcome

**Figure 1:**
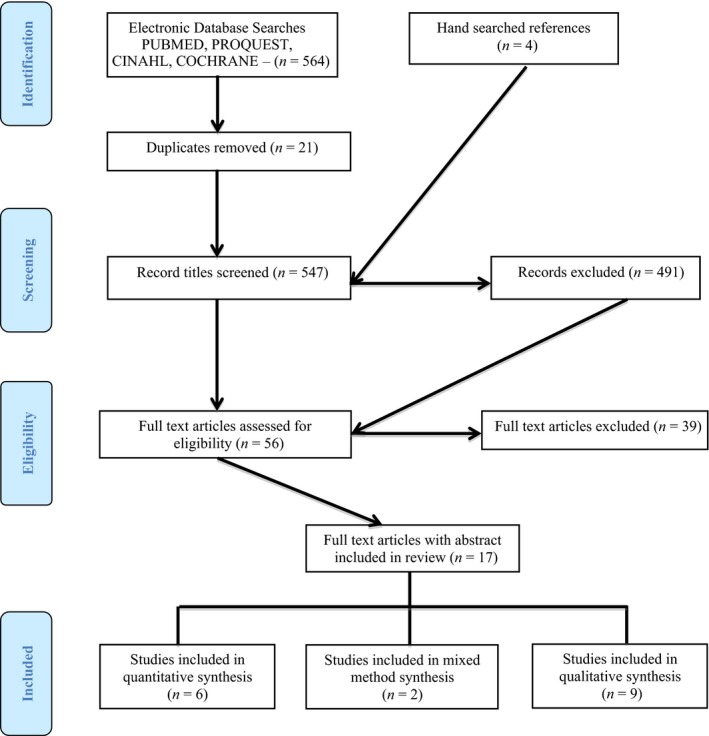
PRISMA diagram of the search strategy.

The initial search outcome generated 564 studies. After 21 duplicates were excluded, the titles and abstracts of these studies were retrieved and read. DM and VA screened the title and abstract of each article. If initial screening indicated the paper was suitable for inclusion, the whole manuscript was read. If any doubt or uncertainty existed, the third author, WC assessed the paper and the three authors reached consensus. From, initially identifying 568 articles, removal of duplications and the screening process led to 56 potential articles. From those, 17 articles were included in the review (Figure 1). Based on the ‘Preferred Reporting items for Systematic Reviews and Meta‐analyses: the PRISMA statement’ (Liberati *et al*. 2009), Figure 1 shows the flow of information through different phases of the review. The PRISMA statement was used to structure the review and systematically report findings (Liberati *et al*. 2009) Figure 1.

### Data extraction and evaluation

When methodologically diverse primary sources are included it increases the complexity of data evaluation (Whittemore & Knafl 2005), thus, careful examination of each study was required in this review. The included studies were first, summarized in tabular form and second, quality appraised to aid data synthesis. Data from the studies relating to approach, context, sample and key findings were extracted. Quality scores were calculated using the Mixed Methods Assessment Tool (MMAT). This scoring system assesses qualitative, quantitative experimental, quantitative observational and mixed methods research studies (Pace *et al*. 2012). The MMAT was used to concomitantly evaluate the quality of studies using various methodologies and to establish their comparative validity and reliability (Pace *et al*. 2012). Tobiano *et al*. (2015), also used the MMAT in their integrative review to assess the quality of the literature. Studies were assessed against the appropriate MMAT criteria based on the methodology used and were assigned quality scores ranging from 0% representing no criteria met, through to 100% representing all criteria met (Pace *et al*. 2012). The MMAT enables the concomitant appraisal of three methodological domains: mixed, qualitative and quantitative. Two researchers, DM and VA independently appraised each article and then compared and discussed their MMAT scores. The third author, WC was available to adjudicate when discrepancies occurred. Quality scores were not used to exclude studies as all studies met at least two criteria, but instead to identify the potential contribution of each study to the overall findings.

### Data analysis

Data were grouped into two domains; recognizing and responding to deterioration and then thematic analysis was used to identify the emerging themes. Findings from each study were coded inductively (Whittemore 2008). This involved reading and rereading the study findings, grouping similar findings into codes and giving each emerging code a label. All codes were subsequently listed and analysed for commonalities and differences. Various findings from one study could be grouped into several different codes. These codes were then reviewed several times to identify themes. A theme was defined as a key characteristic of recognizing and responding to patient deterioration. Initial themes were reorganized based on levels of abstraction until a clear discrimination between themes was evident. This included a creative process of comparing and contrasting displayed data, codes and initial themes to discern commonalities and contradictions in ward nurses' recognition and response to patient deterioration. The process of identifying common themes and relationships by sorting data into groups and orientating ideas gave clarity and focus to the data (Miles & Huberman 1994).

## Findings

Qualitative methodologies were used in nine of the included studies (Table 2), quantitative methodologies in six of the studies (Table 3) and in two studies, mixed methodologies were used (Table 4). Seven of the reviewed studies were conducted in Australia, four in the UK; three in the US and one study was conducted in Singapore, Greece and the Netherlands, The MMAT score of the quantitative studies varied from 25% (Copper *et al*. 2011) to 100% (Mitchell *et al*. 2010, Pantazopoulos *et al*. 2012, Ludikhuize *et al*. 2012).

**Table 2:** Qualitative study characteristics

Author, year country	Aim	Sample	Research design	Rigor, reliability, validity	Analysis	Findings	MMAT score
Andrews and Waterman (2005). UK.	Explore how staff use vital signs and warning signs to package deterioration and respond appropriately.	44 participants (30 RNs, 7, Drs, 7 HCAs). Theoretical sampling.	Grounded theory, interviews and participant observation.	Not identified.	Grounded theory. Open and selective coding.	Three categories emerged: Making credible Grabbing attention Packaging deterioration.	50%
Chau *et al*., (2013). Singapore.	Explored the experiences of EENS with deterioration patients in rep‐cardiac arrest situation and identify strategies to enhance EENs role in caring for deteriorating patients.	15 EENs who had cared for a deteriorating ward patient. One acute hospital in Singapore.	Qualitative. Exploratory descriptive study. Semi‐structured interviews.	Issues related to rigour explored.	Critical incident technique.	Five themes emerged from the data: Recognizing deterioration. Responding to deterioration. Taking responsibility. Educational developments. Modifying clinical processes.	100%
Cioffi (2000). Australia.	Describe the experiences of RNs calling for emergency assistance.	32 female RNs. Four wards in a teaching hospital and three wards in a peripheral hospital in an Australian health service.	Qualitative, exploratory descriptive study. Unstructured interviews.	Two nurse experts examined the interview transcripts for ‘fittingness’	Thematic analysis.	Five main categories: Uncertainty with calling. Identification of change in patient's condition. Identification of at risk situations. Associated feelings. Valuing the MET.	50%
Cox *et al*. (2006). UK.	Explore the influential factors surrounding the experience of trained nurses caring for critically ill patients on general wards.	Seven RNs with a range of experience. One medical ward.	Qualitative. Exploratory descriptive study interview.	Not identified	Content analysis.	Five themes: Clinical environment. Professional relationships. Patient assessment. Feelings. Education needs.	75%
Donohue & Endacott (2009). UK.	Examine processes use during patient deterioration.	11 nurses who had managed a deteriorating patient. Three members of the outreach team.	Qualitative, critical incident technique. Semi‐structured interviews.	Not identified.	Thematic analysis.	Four themes: Individual nature of initial assessment. The use of MEWs to communicate deterioration. Action taken when the patient deteriorated. Team process.	25%
Endecott & Westley (2006). Australia.	Examine the strategies used by nurses to manage patients at risk of deterioration in small rural hospitals.	20 RNs completed questionnaire. Seven RNs interviewed.	Qualitative Case studies	Not identified.	Content analysis.	50% of survey sample was the first to identify deterioration. Three factors depend on effective management of deteriorating patient: Clinical skills. Communication strategies. Rural context.	75%
Gazarian *et al*. (2010).US.	Describe the cues and factors that influence the decision‐making process used by nurses when identifying and interrupting a potential cardiac arrest in the acute care setting.	13 female RNs on four medical wards, who are cared for patients who had experienced a periarrest.	Qualitative, descriptive study. Critical decision‐making method.	Not identified	Cognitive task analysis	Cues used to identify patients at risk: LOC Oxygen status Systolic B/P Knowledge of the patient Factors that influenced RNs decision to interrupt an adverse event. Nursing characteristics: Previous experience of peri‐arrest situations. Ability to function as part of a team. Organizational characteristics: Monitoring equipment. Consultation with more experienced staff. Knowing and valuing team members. Access to knowledge resources.	25%
Massey *et al*. (2014). Australia.	Explore nurses’ experiences and understanding of using and activating a MET.	Three medical wards, one hospital RNs (*n = *15).	Interpretive descriptive.	Transparent audit trail. Creditability and trustworthiness of data confirmed.	Thematic analysis	4 themes from the data: Sensing clinical deterioration. Resisting and hesitating. Pushing the button. Leadership and support.	100%
Minick and Harvey (2003). US.	Describe the early recognition skills of medical/surgical nurses.	14 medical surgical nurses. One hospital in Southern US.	Hermeneutic phenomenological design. Focus group interviews.	Not identified.	Thematic analysis.	Three themes emerged from the data: Knowing the patient directly. Knowing the patient through the family. Knowing something is not as expected.	50%

RNs, registered nurses; MET, medical emergency team; T&T, track and trigger; Drs, Doctors; HCAs, health care assistants; LOC, level of consciousness; BP, blood pressure; EENs, endorsed enrolled nurses; GCS, glasgow coma score; MAEs, MAJOR adverse events; RRS, rapid response systems; SAs, situational awareness; R&R, rural and remote; UNs, unregistered nurses; CRT, capillary refill time; VS, vital signs; ICU, intensive care unit; UN, undergraduate nurses.

**Table 3:** Quantitative study characteristics

Author, year country	Aim	Sample	Research design	Rigor, reliability, validity	Analysis	Findings	MMAT score
Cooper *et al*., (2011). Australia.	To examine in stimulated environment the ability of rural nurses’ to assess and manage patient deterioration.	RNs working in an Australian rural hospital. Participants worked in medical surgical wards (*n = *35).	Exploratory quantitative survey.	Questionnaire previously validated. Previously piloted with student nurses.	Descriptive statistics, Spearman rank order correlation, Pearson correlation coefficients, Wilson *et al*. (1995) signed rank tests and repeated measures *t*‐tests.	Respiratory rate and CRT were the most under assessed VS. Knowledge and management of deterioration varied considerably (27‐91%) mean score = 67%. SA and skill scores were low. Systematic assessment not used. Single vital signs used. Anxiety increased as patient deteriorated and this appeared to affect performance.	25%
Cooper *et al*. (2013). Australia.	To assess the ability of RNs to manage deteriorating patients.	Convenience sample. RNs (*n = *44) 2 hospital wards.	Quasi‐experimental design. Pre‐ and post intervention. The intervention involved 2‐hour session, completion of a demographic survey, completion of a short formative multiple choice questionnaire and team based 8 min videoed scenarios. Assessments and observations were performed to evaluate nurses’ simulated clinical performance.	Questionnaire piloted in two studies, good face and content validity demonstrated.	Descriptive statistics, Spearman rank order correlation, Pearson correlation coefficients, Wilson *et al*. (1995) signed rank tests and *t*‐tests.	Younger nurses scored higher in knowledge. RNs anxiety increased as the patient deteriorated and this affected RNs performance. SA was generally low (median = 50%). SA was higher in younger RNs. Teamwork ratings averaged 57% with a significant association with leadership. Following the intervention participants indicated significant improvements in knowledge, confidence and competence.	75%
Hart *et al*. (2014) US.	To explore and understand medical surgical ward nurses’ perceived self‐confidence and leadership abilities as first responders in recognizing and responding to patient deterioration.	Convenience sample of registered medical surgical nurses (*n = *148) Five hospitals in the US.	Prospective, cross‐sectional descriptive, quantitative survey.	Self‐confidence scale and leadership ability questionnaire, Internal consistency of the tool has been previously demonstrated. Validity of the instruments not identified.	Descriptive statistics, Pearson's correlation and regression analysis.	RNs reported moderate self‐confidence in recognizing, assessing and intervening during clinical deterioration. RNs reported moderate self‐confidence when performing leadership skills prior to the arrival of the RRS. A significant positive relationship was found between self‐confidence and leadership abilities. Age and certification status were significant predictors of nurses’ leadership ability.	75%
Ludikhuize *et al*. (2012). Netherlands.	Describe how nurses and doctors judge the quality of care while caring for deteriorating patients on medical wards, compared with the judgement of independent experts.	Convenience sample: Nurses (*n = *49) Residents (*n = *36) Specialist (*n = *32).	Cross‐sectional study using interviews of care providers compared with retrospective judgements of independent experts. 47 major adverse events and 198 interviews were analysed.	Literature review and cross‐referencing of key publications and peer review were used to identify five domains involved in hospital settings. Domains used to develop the survey. Face validity was tested on three nurses and two residents. Interobserver correlation (Cohen's k) was calculated.	Mean medium and IQR ranges. Kruskal–Wallis used to test 3 or more groups. Friedman test, Wilcoxon signed rank tests used to test for differences.	Care providers rate their care in the hours preceding a MAE as good. Communication, cooperation and coordination were graded positively. Medical staff graded these factors higher than nurses. Independent experts were more critical of care provided.	100%
Mitchell *et al*., (2010). Australia.	To determine whether the introduction of multifaceted intervention to detect clinical deterioration in patients would decrease the rate of predefined adverse outcomes.	Two Australian hospitals. Four study wards. Two from each hospital. Mixed medical surgical wards. The intervention incorporated a newly designed ward observation chart, T&T tool, and an education programme. 177 ward nursing staff, 28 junior medical officers and five physiotherapists undertook an Educational programme.	Prospective controlled before and after intervention trial.	Regression used to adjust for confounding. No information on data collection or how patient's charts were accessed or analysed.	Descriptive statistics. Chi‐Square and logistic regression, Negative binominal regression and Log rank test.	Decrease in unexpected admission to ICU. Significant increase in the number of patients receiving one or more MET reviews. Increase in length of stay. Decrease in unexpected deaths Increase in documentation of VS.	100%
Pantazopoulos *et al*., (2012) Greece.	Evaluate the relationship between nurse demographics and correct identification of clinical situations that warrant MET activation.	94 nurses (response rate of 62%).	Descriptive quantitative survey.	Test–retest procedure was used to test reliability. 10% of the previously surveyed respondents were randomly selected to complete the survey 1 week later. Level of answer agreement was 93%. Internal consistency of the survey was measured using Cronbach's alpha coefficient. The coefficient was 0·78.	Descriptive statistics. Mann–Whitney to compare knowledge scores between the two groups.	Nurses (with 4 year degree) identified clinical deterioration more accurately. Nurses educated with resuscitation techniques were more able to treat deterioration correctly. Physiological parameters are important in responding and recognizing deterioration. Respiratory rate and GCS were the least assessed vital signs.	100%

RNs, registered nurses; MET, medical emergency team; T&T, track and trigger; Drs, Doctors; HCAs, health care assistants; LOC, level of consciousness; BP, blood pressure; IQR, interquartile range; EENs, endorsed enrolled nurses; GCS, glasgow coma score; MAEs, MAJOR adverse events; RRS, rapid response systems; SAs, situational awareness; R&R, rural and remote; UNs, unregistered nurses; CRT, capillary refill time; VS, vital signs; ICU, intensive care unit; UN, Undergraduate Nurses.

**Table 4:** Mixed method study characteristics

Author, year country	Aim	Sample	Research design	Rigor, reliability, validity	Analysis	Findings	MMAT score
Endacott *et al*. (2007) Australia.	Identify the cues that ward nurses and doctors use to identify deterioration. Examine assessment and communication of deterioration.	RNs (*n = *11) Drs (*n = *14) Chart audit (*n = *17)	Case study,	Not addressed.	Interviews analysed via content analysis and chart via descriptive statistics.	RNs & Drs relied on vital signs to identify patient deterioration. RNs relied on patient's physical assessment of patient capabilities, compared to Drs who undertook additional clinical investigations. Admission category and co‐morbidities increased clinicians’ identification of deterioration. There was a lack of timely referral to more senior clinicians. GCS and urine output was not charted at all.	75%
McDonnell *et al*., (2012) UK.	Evaluate the impact of a new T&T and observation chart on the knowledge and confidence of nurses to recognize & manage deteriorating patients..	Study conducted on 12 wards. Survey (*n = *212) and qualitative interviews (*n = *15) with RNs & UNs, 6 weeks before and after an intervention. The intervention included training, the introduction of a new T&T system and the introduction of a new observation chart.	A single centre, mixed methods, before and after study	The questionnaire based on existing instruments with established face and content validity. Questionnaire was piloted. Qualitative analysis performed by one researcher, independent analysis of a sample of transcripts undertaken.	Descriptive statistic, *t*‐tests and McNemar tests. Interview data was analysed using thematic analysis.	66% paired response rate. Following the intervention the numbers of staff concern were significantly reduced. Staff knowledge and confidence significantly increased following the intervention. UNs scores improved more than RNs. Three themes emerged from the data: Staff concerns. Staff knowledge. Confidence and differences between RNs and UNs.	100%

RNs, registered nurses; MET, medical emergency team; T&T, track and trigger; Drs, Doctors; HCAs, health care assistants; LOC, level of consciousness; BP, blood pressure; EENs, endorsed enrolled nurses; GCS, glasgow coma score; MAEs, MAJOR adverse events; RRS, rapid response systems; SAs, situational awareness; R&R, rural and remote; UNs, unregistered nurses; CRT, capillary refill time; VS, vital signs; ICU, intensive care unit; UN, undergraduate nurses.

The mixed method studies scored between 75% (Endacott *et al*. 2007) and 100% (McDonnell *et al*. 2013). The methodological quality of the studies varied in the qualitative studies, with two studies (Massey *et al*. 2009, Chau *et al*. 2013) meeting 100% of the MMAT quality criteria and two studies meeting 25% of the MMAT criteria (Donohue & Endacott 2010, Gazarian *et al*. 2010). Attempts to enhance credibility through investigating different ward setting were noted, with most researchers studying more than one ward. In terms of sampling, the majority of the qualitative studies explored registered nurses' experiences of recognizing and responding to patient deterioration. Only one study (Chau *et al*. 2013) explored enrolled nurses' experiences.

Two key domains were identified from the literature (DeVita *et al*. 2006); first, recognizing deterioration and second, responding to deterioration. Both of these domains were closely aligned with the aims of the integrative review. The first domain, recognizing patient deterioration encapsulated four themes: (1) assessing the patient; (2) knowing the patient; (3) education and (4) equipment. The second domain, responding to patient deterioration, was encapsulated in three themes; (1) non‐technical skills; (2) access to support and (3) negative emotional responses.

### Recognizing patient deterioration

Recognition of patient deterioration was underpinned by four themes: (1) assessing the patient; (2) knowing the patient; (3) education and (4) equipment.

#### Assessing the patient

Assessing the patient was identified as a significant theme in recognizing patient deterioration. Nine studies identified that assessment of the patient played an important role in enabling nurses to recognize patient deterioration in a timely fashion (Minick & Harvey 2003, Andrews & Waterman 2005, Cox *et al*. 2006, Endacott & Westley 2006, Endacott *et al*. 2007, Gazarian *et al*. 2010, Pantazopoulos *et al*. 2012, Chua *et al*. 2013, Massey *et al*. 2014). Vital signs and observations were identified as particularly important in assessing the patient and recognizing patient deterioration. Nurses commonly reported that changes in the patient's vital signs or observations were quantifiable indicators that the patient was deteriorating. Nurses used changes in patients’ vital signs to ‘package’ deterioration to medical staff so that care could be escalated (Andrews & Waterman 2005). Vital signs were used as cues to recognize timely deterioration and assist in the decision‐making process in relation to escalating care for the patient (Gazarian *et al*. 2010).

#### Knowing the patient

Knowing the patient was identified as a key theme in recognizing patient deterioration in five studies (Cioffi 2000, Minick & Harvey 2003, Andrews & Waterman 2005, Cox *et al*. 2006, Gazarian *et al*. 2010). Often, familiarity with the patient was linked to awareness of very subtle changes in the patient status. Nurses recognized patient deterioration through a heightened familiarity of the patient's medical history (Minick & Harvey 2003). Ward nurses also recognized patient deterioration through ‘gut feelings or a sixth sense’ and identified this as intuition (Cioffi 2000, Cox *et al*. 2006, Massey *et al*. 2014). Ward nurses then used these subtle cues to recognize that the patient was deteriorating and not knowing the patient acted as a barrier to recognizing deterioration (Gazarian *et al*. 2010).

#### Education

Education was identified as an important factor in recognizing patient deterioration in five studies (Cox *et al*. 2006, Pantazopoulos *et al*. 2012, Chua *et al*. 2013, McDonnell *et al*. 2013, Hart *et al*. 2014). Ongoing specific clinical education and skills training was identified as imperative in enabling nurses to recognize and respond to patient deterioration (Cox *et al*. 2006, McDonnell *et al*. 2013). The level of education was identified as a significant predictor in ward nurses' ability to promptly recognize patient deterioration (Pantazopoulos *et al*. 2012). Nurses who had graduated from a 4‐year university educational programme identified patient deterioration significantly quicker than nurses who had graduated from a 2‐year educational programme (Pantazopoulos *et al*. 2012). Nurses who had obtained a postgraduate qualification were more self‐confident in recognizing patient deterioration.

#### Equipment

The use of specialized equipment influenced registered nurses ability to recognize timely patient deterioration (Cox *et al*. 2006, Gazarian *et al*. 2010). Cox and colleagues thought nurses relied on machinery and equipment to the detriment of holistic patient assessment and this impeded and delayed recognition of deterioration. In contrast, Gazarian *et al*. (2010) highlighted that nurses valued the use of equipment and frequently reported using equipment to aid and assist in timely recognition of patient deterioration. Unfamiliarity with equipment hindered nurses’ ability to recognize patient deterioration (Cox *et al*. 2006).

### Responding to patient deterioration

Three themes were identified as important in assisting ward nurses to successfully respond to patient deterioration: (1) non‐technical skills; (2) access to support and (3) negative emotional responses.

#### Non‐technical skills

Thematic analysis of the research identified that effective, leadership, teamwork, communication and situational awareness enabled nurses to more effectively respond to the deteriorating patient. These three criteria are often defined as non‐technical skills (Endsley 1995, Flin *et al*. 2008, Stubbings *et al*. 2012). The importance of non‐technical skills in supporting ward nurses to respond to patient deterioration was reported in eight studies (Andrews & Waterman 2005, Cox *et al*. 2006, Donohue & Endacott 2010, Gazarian *et al*. 2010, Ludikhuize *et al*. 2012, Cooper *et al*. 2013, Hart *et al*. 2014, Massey *et al*. 2014). Nurses who executed strong leadership abilities were more confident about responding to the deteriorating patient (Hart *et al*. 2014). Effective communication skills including the use of appropriate medical language (Andrews & Waterman 2005) resulted in a positive response to patient deterioration. A supportive team was also identified as an essential element in responding to patient deterioration (Cox *et al*. 2006, Gazarian *et al*. 2010). Non‐technical skills were identified as imperative because they promoted a more structured and organized response to patient deterioration.

#### Accessing support

Nurses' access to support from medical and nursing colleagues was identified as important in six studies (Cioffi 2000, Andrews & Waterman 2005, Cox *et al*. 2006, Donohue & Endacott 2010, Gazarian *et al*. 2010, Massey *et al*. 2014). Ward nurses often required help and support in recognizing and responding to patient deterioration, frequently seeking this support from peers or more senior nurses, or medical staff (Massey *et al*. 2014). The ability to ‘grab attention’ (Andrews & Waterman 2005) linked to effective communication skills, confidence and level of experience and experience (Cioffi 2000, Massey *et al*. 2014). Ward nurses actively sought consultation with more experienced nurses. This consultation was linked to a sense of mutual respect and trust. When ward nurses did not know other team members a delay in responding to patient deterioration ensued (Gazarian *et al*. 2010).

#### Negative emotional responses

Six studies reported that responding to patient deterioration was associated with negative emotional responses (Cioffi 2000, Andrews & Waterman 2005, Cox *et al*. 2006, Cooper *et al*. 2013, Massey *et al*. 2014). Feelings of anxiety, fear and panic were reported in three studies (Cioffi 2000, Cox *et al*. 2006, Massey *et al*. 2014). Ward nurses feared looking stupid, being reprimanded or being ridiculed when responding to the deteriorating patient (Andrews & Waterman 2005) and also felt their professional creditability could be threatened. These negative emotions delayed escalation of care for deteriorating patients (Massey *et al*. 2014).

## Discussion

In this integrative review, we identified, described and analysed the factors impacting on ward nurses’ ability to recognize and respond to patient deterioration reported in the literature. Previous literature reviews on patient deterioration have focused on new graduate nurses' response and recognition to patient deterioration (Purling & King 2012) and educational strategies to improve nurse's recognition of patient deterioration (Liaw *et al*. 2011). The concept of recognizing and responding to patient deterioration is an internationally important clinical topic as demonstrated by the international literature accessed and synthesized in this review.

This is the first integrative review to explore ward nurses recognition and response to patient deterioration. It is also the first integrative review to use the recently developed definition of clinical deterioration (Jones *et al*. 2013). Using a clearly defined definition of deterioration enabled a focused and succinct review and we have also demonstrated the usefulness of using this definition in future patient deterioration research. Limitations of the reviewed studies include small sample sizes, single locations and minimal discussion of the reliability, validity or rigor of the study. Although inclusion of the studies in this integrative review was justified because of the limited research available on this topic. The strengths of the quantitative studies included use of a validated tool (Ludikhuize *et al*. 2012,.), multiple sites (Mitchell *et al*. 2010) and strategies used to enhance reliability and validity (Table 2). The MMAT score was lower for the study with a small sample and a data collection tool that had been validated for a different population (Cooper *et al*.^2011^).

Our findings indicate ward nurses' experience and negotiate considerable clinical, organizational and system barriers in relation to recognizing and responding to clinical deterioration. This finding is reflected in previous research (Odell *et al*. 2009, Johnston *et al*. 2015, Osborne *et al*. 2015), suggesting that these challenges and issues continue to exist for nurses.

The concept of recognizing and responding to patient deterioration has emerged primarily from the critical care arena, with minimal overlap or acknowledgement of nursing health services systems and structures. For example, the contribution of the registered nurse workforce to patient outcomes has been a major focus for several well‐known international groups of researchers (Aiken *et al*. 2002, Hall *et al*. 2004, Tourangeau *et al*. 2007) and the findings of their work continues to impact on nurse patient ratios. Despite, this our inclusion criteria and search strategy did not identify this important research. The critical care community have adopted and use the terms ‘recognizing’ and ‘responding’ to deterioration while, nursing health service researchers have defined and use terms such as ‘failure to rescue’ and nurses’ surveillance capacity (Kutney Lee *et al*. 2009). The lack of Nomenclature creates challenges when identifying inclusion and exclusion criteria and search terms for literature reviews. Potentially, new insights or theories on the topic may be missed.

Ward nurses’ role in recording and documenting vital signs means they are ideally placed to recognize and respond to deteriorating patients (Aiken *et al*. 2002, Clarke 2004, Osborne *et al*. 2015) and therefore they must be able to undertake physical assessment effectively and escalate patient care needs accordingly (Douglas *et al*. 2014). Recognizing and responding to the deteriorating patient is complex, challenging and multifaceted. Confounding propositions regarding the factors that contribute to ward nurses' recognition and response to the deteriorating patients exist in the literature (Odell *et al*. 2009, Johnston *et al*. 2015). It is well‐known, that patient safety is compromised when a delay occurs in escalating care in response to clinical deterioration (Johnston *et al*. 2015). The reasons for these delays are complex and poorly understood. This integrative review adds to the existing knowledge of the topic. It suggests potential strategies such as education, creating a just culture and the effective use of non‐technical skills could be implemented to improve the nursing care and management of the deteriorating patient.

We identified four themes in this integrative review that promoted or impeded the recognition of patient deterioration by ward nurses and thus delayed appropriate and timely escalation of care. First, patient assessment, the recording and documentation of vital signs were acknowledged as crucial in supporting ward nurses to recognize patient deterioration, this important finding has also been observed by other researchers (Douglas *et al*. 2014, Osborne *et al*. 2015). Recognition of physiological abnormalities is primarily a nursing role (Clarke 2004, Considine 2005, Massey & Meredith 2010) and nurses are responsible for assessment, recording and documenting of vital signs. Recently, however, there has been increasing concern that recording and documenting vital signs, often referred to in clinical practice as; ‘doing the obs’, has become reliant on technology, rendered ritualistic and task‐oriented, a passive process often delegated to most junior staff (Wheatley 2006, James *et al*. 2010, Douglas *et al*. 2014). Some researchers argue that failure by nurses to appreciate the importance of vital signs leads to a loss of detailed and holistic patient assessment (Douglas *et al*. 2014, Osborne *et al*. 2015); this delays appropriate treatment and significantly compromises patient safety. This finding, suggests the need for a significant paradigm shift. Clinical nurses need to move away from ‘doing the obs’ to performing a holistic assessment of the patient, documenting the findings of this assessment and when appropriate initiating appropriate escalation protocols.

Knowing the patient was identified in this integrative review as an important factor in recognizing patient deterioration, Odell *et al*. (2009) systematic review also supports this finding and highlighted the importance of nurses’ use of intuition in responding to patient deterioration. Knowing the patient enabled ward nurses to recognize subtle changes in the patient's condition. Knowledge of the patient enables and facilitates the correct interpretation of vital signs and physiological indicators in the context of each patient and their medical history, thus promoting holistic patient assessment (Morrison & Symes 2011). Knowing the patient led to a sense of salience and an ability to recognize aspects of the patient's clinical situation that stand out as important when guiding ward nurses' judgment (Benner & Tanner 1987). We identified in this integrative review, that ward nurses acknowledged the importance of information gained from observing a patient, interpreting physiological parameters, knowing the patient and looking at and questioning previous data to provide an overall picture of the patient. Thus, the ability to use both objective criteria, for example, data generated from the vital signs and subjective criteria, such as knowing the patient were important in recognizing patient deterioration. Knowing the patient was often linked to a sixth sense, a feeling that something was not quite right or intuition and these subjective feelings were developed from experience. However, the importance of experience in relation to recognizing clinical deterioration was not consistent with the literature (Ericsson 2008). Ericsson *et al*. (2007), Greenwood and King (1995) argue that expertise and experience are unrelated. What does appear to improve nurses’ ability to recognize clinical deterioration is not simply the product of experience but of deliberate clinical practice (Minick & Harvey 2003, Ericsson *et al*. 2007). Deliberate practice, or: ‘deliberate efforts have been defined as the desire to improve one's performance beyond its current level’ (Ericsson, 2007, p. 991). As recognition and response to patient deterioration requires the identification and synthesis of multiple cues from patients, successful recognition and response to patient deterioration requires ward nurses constantly improve, refresh and develop their knowledge and skills in this complex clinical area. Ward nurses, therefore, need support and guidance from supervisors, team leaders and educators to identify areas of their practice and performance that can be improved.

Education was identified in this review as an important factor in enabling nurses to recognize clinical deterioration (Cox *et al*. 2006, Pantazopoulos *et al*. 2012, Chua *et al*. 2013, McDonnell *et al*. 2013, Hart *et al*. 2014) and is also supported by other researchers (Douglas *et al*. 2014, Odell *et al*. 2009, Purling & King 2012). What was not clear in this integrative review was the amount and type of education that ward nurses felt helped them to recognize patient deterioration. Increasingly, deliberate practice in the form of simulation has been gaining popularity in the undergraduate and postgraduate nursing curriculum (Fisher & King 2013, Hart *et al*. 2014). Fisher and King (2013) discuss how simulation facilitated learning in a safe environment and improved nursing students’ clinical skills in recognizing and responding to patient deterioration, thus improving patient care and safety. The use of simulation to improve ward nurses' performance and develop deliberate practice skills has not been evaluated and there is an urgent need for this deficit to be addressed. We also identified that nurses who had completed a postgraduate qualification were more confident in recognizing and responding to patient deterioration (Pantazopoulos *et al*. 2012), suggesting that formal university qualifications may be more appropriate in improving ward nurses recognition of patient deterioration rather than hospital based in‐service study days or session.

Over reliance on technology and equipment impeded ward nurses ability to recognize patient deterioration (Cox *et al*. 2006, Gazarian *et al*. 2010) because nurses tended to use technology rather than perform holistic patient assessments and this often delayed recognition of deterioration, Odell *et al*. (2009), also argue that technology impacts on nurses’ ability to respond to patient deterioration. Technology will play an increasingly important role in the recognition of clinical deterioration. Patient surveillance systems (PSS) (Sahandi *et al*. 2010) have been developed to improve recognition of patient deterioration. PSSs use continuous patient vital sign monitoring in the general care setting to facilitate early recognition of patient deterioration (Nangalia *et al*. 2010). PSSs are gathering momentum in the clinical area and future research will assess these systems ability to safely and appropriately recognize patient deterioration.

We identified three themes in this integrative review that promoted or impeded ward nurses response to patient deterioration even once it was identified/recognized. First, non‐technical skills were identified as key in enabling ward nurses to respond effectively and promptly to patient deterioration (Andrews & Waterman 2005, Cox *et al*. 2006, Donohue & Endacott 2010, Gazarian *et al*. 2010, Ludikhuize *et al*. 2012, Cooper *et al*. 2013, Hart *et al*. 2014, Massey *et al*. 2014). A recent systematic review (Johnston *et al*. 2015) also found that non‐technical skills were a significant factor in promoting nurses timely responses to patient deterioration (Johnston *et al*. 2015). In this review, we found ward nurses were better positioned to respond to patient deterioration when they knew and trusted the team they were working with. This familiarity made them more confident in responding to patient deterioration Non‐technical skills have been identified as important in reducing adverse events and promoting patient safety in a variety of specialized clinical settings (Stubbings *et al*. 2012, Johnston *et al*. 2015).

Accessing support was the second theme influencing ward nurses ability to respond to patient deterioration, this finding is also consistent with other research (Purling *et al*. 2012, Johnston *et al*. 2015). In this integrative review, we found ward nurses access to support and advice delayed timely response to patient deterioration and this impacted negatively on patient outcomes. Ward nurses were more likely to seek advice or confirmation from their peers or more senior nurses. This finding is concerning since literature highlights that experience does not correlate with nurses ability to respond to patient deterioration (Ludikhuize *et al*. 2012, Hart *et al*. 2014, Douglas *et al*. 2014). This practice may delay appropriate clinical management, escalation of care and jeopardize patient safety. Bagshaw and colleagues reported that nurses appeared to prefer to access help or support from among their team and ‘use the home team’ rather than escalating patient care needs (Bagshaw *et al*. 2010). A ward nurse may identify clinical deterioration in a patient; then, when the clinical situation is deemed beyond the expertise of the ward nurse, ask a more senior nurse for advice. Then, a junior doctor is consulted who responds based on their skills and their knowledge of the situation. When the junior doctor's knowledge and skills are exhausted, another call is made, then another and another, until all available resources have been exhausted. This knowledge and skills ladder is clearly hierarchical in nature and contributes to the delay in escalation of care.

The final theme in this integrative review that influenced ward nurses response to patient deterioration was the emotional feelings responding to patient deterioration engendered. Others assert that negative emotional feelings deterred nurses from responding to patient deterioration (Johnston *et al*. 2015, Odell *et al*. 2009,. In this integrative review, we found ward nurses were anxious about making the wrong decision and looking foolish or stupid, and these feelings delayed response to patient deterioration. This finding confirms that recognizing patient deterioration is an emotionally charged experience that can incite panic, anxiety and fear (Considine 2005, Shapiro *et al*. 2010, Johnston *et al*. 2015). Although there is general consensus that a ‘no blame’ culture is an important element in successful recognition and response of patient deterioration this important message may not be translated into clinical practice. The culture of the ward and the organizations clearly serves as an important facilitator for successful recognition and response to patient deterioration by ward nurses. Nurse managers, educators and supervisors need to promote a clinical culture that ensures ward nurses feel supported when responding to patient deterioration.

### Limitations

While rigorous methods were used for this review, there are limitations. While the search was exhaustive and robust, some of the published research may have been missed and as such; this review may not be representative of all relevant work in the field. While the MMAT has established validity its reliability and validity in various reviews may differ.

### Recommendations for practice

Knowing the patient was identified in this integrative review as a significant factor in recognizing patient deterioration. This phenomenon indicates the importance of nurse specialization. If patients with specific disease processes were admitted to specialist wards this would develop nurses’ expertise in caring for similar types of patients. This model of care would require significant support from hospital managers, clinicians and medical staff. Alternatively, the findings of this review suggest that rather than specialized wards or clinical areas a specialized area of practice in the area of recognizing and responding to patient deterioration be developed. The emergence of high capability rapid response teams (DeVita *et al*. 2006) is indicative that this model is the favoured paradigm currently being implemented internationally.

Activities that promote collaborative practices and a ‘just’ culture need to be developed in clinical practice to offset the hierarchal nature of clinical practice, because this negatively impacts on ward nurses’ abilities to respond to patient deterioration Therefore, strategies that promote positive team working and develop non‐technical skills should be implemented to reduce the anxiety associated with responding to patient deterioration, so that a culture of patient safety can be developed in the ward environment. An improved culture of safety has been linked with fewer incidents of errors in patient safety and better outcomes (Johnston *et al*. 2015). Hospital managers and leaders need to explore and implement strategies and solutions that minimize ward nurses experiences of negative emotions when responding to patient deterioration.

### Recommendations for research

The findings from this integrative review highlight the need for further research into this important clinical area. Physiological parameters or vital signs were identified in this review as being one of the most important predictors ward nurses used to recognize and respond to clinical deterioration. However, there are no studies that explore how nurses use vital signs to recognize and respond to patient deterioration or how effective ward nurses are at assessing patients’ vital signs. Future research needs to explore this important area. Non‐technical skills were also identified as important in promoting or impeding nurses’ response to patient deterioration. The importance of non‐technical skills has been explored in other clinical areas (Stubbings *et al*. 2012) but research examining the importance of non‐technical skills in relation to recognizing and responding to patient deterioration is lacking.

### Recommendations for education

An important factor in enabling nurses to recognize clinical deterioration in this review was education. The Acute Life‐threatening Events: Recognition and Treatment (ALERT) course (Smith & Poplett 2002, 2004) used deliberate practice in the form of stimulation to improve medical staffs’ knowledge and performance in recognizing and responding to the deteriorating ward patient. Simulation and courses that use deliberate practice techniques should be explored as a potential strategy that could be implemented in the clinical setting to improve ward nurses recognition of patient deterioration. A clinical reasoning educational model proposed by Levett‐Jones *et al*. (2010) could be useful for ward nurses caring for patients at risk of deteriorating. Specific physiological measurements have been identified as significant early warning signs in identification of deterioration and these should be could be emphasized and taught to ward nurses (Levett‐Jones *et al*. 2010, Purling *et al*. 2012).

## Conclusion

The value of ward nurses’ ability to recognize and respond to patient's deterioration, reduce adverse events and promote patient safety cannot be understated. In this integrative review, we have confirmed that the recognition and management of the deteriorating patient is complex and multidimensional. Patient acuity will continue to increase in hospital wards as the inpatient population becomes older and sicker with more complex care needs**.** Research, education and health care providers need to ensure that there are educational development and system modifications in place to enhance the ability of ward nurses’ to recognize and respond to patient deterioration.

## Author contributions

All authors have agreed on the final version and meet at least one of the following criteria [recommended by the ICMJE (http://www.icmje.org/recommendations/)]:

- substantial contributions to conception and design, acquisition of data or analysis and interpretation of data;
- drafting the article or revising it critically for important intellectual content.
